# *MYLK* and *PTGS1* Genetic Variations Associated with Osteoporosis and Benign Breast Tumors in Korean Women

**DOI:** 10.3390/genes12030378

**Published:** 2021-03-06

**Authors:** Hye-Won Cho, Hyun-Seok Jin, Yong-Bin Eom

**Affiliations:** 1 Department of Medical Sciences, Graduate School, Soonchunhyang University, Asan, Chungnam 31538, Korea; sandy1024@naver.com; 2 Department of Biomedical Laboratory Science, College of Life and Health Sciences, Hoseo University, Asan, Chungnam 31499, Korea; jinhs@hoseo.edu; 3 Department of Biomedical Laboratory Science, College of Medical Sciences, Soonchunhyang University, Asan, Chungnam 31538, Korea

**Keywords:** osteoporosis, benign breast tumor, ovariectomy, *MYLK*, *PTGS1*, genome-wide association study

## Abstract

Osteoporosis, characterized by reduced bone mass and increased bone fragility, is a disease prevalent in women. Likewise, breast cancer is a multifactorial disease and considered the major cause of mortality in premenopausal and postmenopausal women worldwide. Our data demonstrated the association of the *MYLK* gene and *PTGS1* gene variants with osteoporosis and benign breast tumor risk and the impact of ovariectomy on osteoporosis in Korean women. We performed a genome-wide association study (GWAS) of women with osteoporosis and benign breast tumors. There were 60 single nucleotide polymorphisms (SNPs) and 12 SNPs in the *MYLK* and *PTGS1* genes, associated with benign breast tumors and osteoporosis. Our study showed that women with homozygous *MYLK* rs12163585 major alleles had an increased risk of osteoporosis following ovariectomy compared to those with minor alleles. Women carrying the minor *PTGS1* rs1213265 allele and not treated via ovariectomy carried a higher risk of osteoporosis than those who underwent ovariectomy with a homozygous genotype at the major alleles. Our results suggest that both the *MYLK* and *PTGS1* genes are genetic factors associated with the phenotypes, and these associations appear to be modulated by ovariectomy.

## 1. Introduction

Osteoporosis is defined by low bone mass and deterioration in bone architecture [1,2]. It is mainly caused by factors such as increasing age, postmenopausal status, deficiencies in sex hormones like estrogen and androgen, premature ovarian failure, ethnic background, low body mass index, and vitamin D deficiency [3]. Previous studies have shown that early or premature menopause and ovarian resection were associated with the risk of osteoporosis due to the effect of estrogen deficiency on osteoclasts [4,5,6]. The Korean National Health and Nutrition Examination Survey (KNHANES) reported that the incidence of osteoporosis in Korean females aged 50 years and older was 38% [7].

Benign breast disease, which proliferates in epithelial tissue, is a breast cancer precursor associated with an increased risk of breast cancer [8,9]. Women carrying benign breast tumors had a two-fold increased risk of breast cancer and a five-fold increased risk of an atypical hyperplasia [8]. Breast cancer is a multifactorial disease and the major cause of mortality in premenopausal and postmenopausal women worldwide [10]. The accumulation of adipocytes in postmenopausal women can influence breast cancer development by increasing estrogen and insulin levels [11,12,13].

Thus, biochemical and genetic links between postmenopausal osteoporosis and breast disease are of great interest. Both bone and breast tissue are regulated not only by estrogen, which is a hormone that controls bone density, but also receptor activator of nuclear factor-κB ligand (RANKL), thereby restoring the equilibrium between bone formation and resorption [14,15,16,17]. In postmenopausal women, breast cancer and osteoporosis are common, and although both are dependent on estrogens this leads to conflicting implications for the diagnosis and treatment, that is, estrogens reduce the risk of fractures but increase the risk of breast cancer. In particular, the RANKL/RANK pathway, regulating osteoclast differentiation and activation, is also involved in breast carcinogenesis [18].

To prevent and treat heavy menstrual bleeding, dysmenorrhea, chronic pelvic pain, endometriosis, uterine prolapse, and gynecologic cancer, ovariectomy, a major gynecologic procedure, has been performed in premenopausal women [4]. Breast cancer and osteoporosis are affected by estrogen levels, which complicate the diagnosis and treatment. Estrogens, which are secreted by the ovary, reduce the risk of fractures but increase the risk of breast cancer [19]. Therefore, ovariectomy prevents breast cancer but is one of the risk factors for bone loss [20]. One study investigated the risk of osteoporosis in Korean women who underwent hysterectomy, which increased the risk of osteoporosis regardless of age or bilateral ovariectomy [4].

To the best of our knowledge, no study has demonstrated an association between both *MYLK* and *PTGS1* genes in the risk of osteoporosis and benign breast tumor. Furthermore, few/no study has examined the impact of ovariectomy on gene-disease risk for osteoporosis among Korean women. We identified the SNPs in our genome-wide association study (GWAS) that simultaneously increased the risk of osteoporosis and benign breast tumors.

## 2. Materials and Methods

### 2.1. Study Population

The present study was performed with data obtained from the Korean Genome and Epidemiology Study (KoGES) [21], which was a large-scale cohort study conducted in a Korean population. KoGES is composed of six types cohorts, including subjects from the Health Examinee (HEXA) study used to determine the association between benign breast tumors, osteoporosis, and ovariectomy. Details of the KoGES and HEXA studies are described elsewhere [21]. Briefly, a total of 173,357 participants aged 40–79 years were recruited between 2004 and 2013. Following informed consent, the participants were examined at health examination centers in Korea. We performed a series of cross-sectional analyses in the present study by the baseline data from the HEXA study [21].

**Figure 1:**
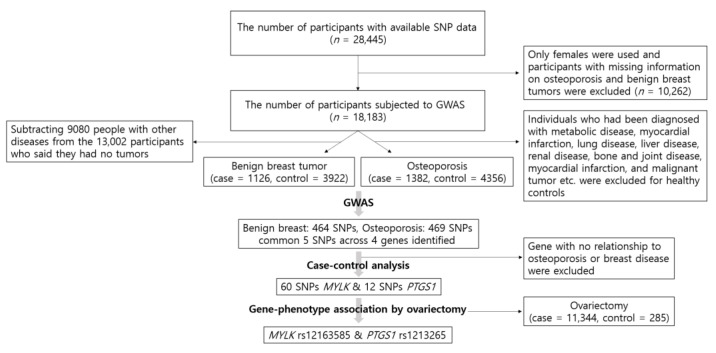
Flow chart of exclusion criteria in the present study population and the process from genome-wide association study (GWAS).

Figure 1 is a schematic illustration depicting the participant selection process for this study and the process from GWAS.

We used GWAS to test the association between SNPs and the genetic risk of benign breast tumors and osteoporosis. Participants with missing information on osteoporosis and benign breast tumors were excluded from the 28,445 participants with accessible SNP information. In addition, since we analyzed the association between osteoporosis and ovariectomy, only females were evaluated in this study (*n* = 18,183). A control group of 6518 healthy participants was used in the present study, which consisted of individuals who were not diagnosed with hypertension, diabetes mellitus, hyperlipidemia, transient ischemic attack, myocardial infarction, chronic gastritis, gastric ulcer, intestinal polyps, acute liver disease, fatty liver, cholelithiasis, chronic bronchitis, chronic obstructive pulmonary disease (COPD), asthma, allergy, thyroid disease, arthritis, osteoporosis, gout, cataracts, glaucoma, periodontal disease, chronic nephritis, renal failure, malignant tumor, or fractures. Since the disease history was included in the KoGES project, it was selected as the criterion for healthy controls. Of the 6518 healthy controls, 2162 males were excluded and a total of 4356 healthy female controls were used. However, because benign breast tumors were not considered when the healthy controls were identified, the healthy control group for benign breast tumors included a final number of 3922 after subtracting 9080 subjects with other diseases from the 13,002 participants who stated the absence of tumors. The diagnosis of benign breast tumors and/or osteoporosis was made by a medical doctor. Consequently, 4356 healthy controls and 1382 osteoporosis cases were identified, and 3922 healthy controls and 1126 benign breast tumor cases were analyzed.

### 2.2. Assignment of Ovariectomy

Information on the ovariectomies was obtained through self-report during a trained interviewer administered survey. The questionnaire was composed of four options: 1 = no, did not undergo ovariectomy, 2 = yes, removed only one ovary, 3 = yes, removed both of them but partially, and 4 = yes, removed both of them entirely. To ensure accurate results, the participants who answered 1–3 were defined as the “no ovariectomy” group and the participants with responses of 4 were defined as the ovariectomy group. Of a total of 18,183 women, 11,344 answered the questionnaire, 11,629 women were defined as the “no ovariectomy” group, and 285 women were defined as the ovariectomy group. The subjects’ age based on a response of 4, indicating both ovaries were entirely removed, was considered the surgical age.

### 2.3. Genome-Wide Genotyping and Selection of SNPs

Genotype data were obtained from the Center for Genome Science, Korea National Institute of Health. DNA samples isolated from the participants were genotyped with an Axiom^®^ 2.0 Reagent Kit (Affymetrix Axiom^®^ 2.0 Assay User Guide). The genotype data were obtained from the K-CHIP designed by the Center for Genome Science at the Korea National Institute of Health. Additional information regarding this protocol has been presented elsewhere [22,23]. Subjects with a high missing call rate (>10%), high missing genotype rates (>5%), minor allele frequency <0.01, or gender inconsistency were excluded during the quality control process. We performed GWAS and selected SNPs with a *p*-value of less than 0.001. The location of the genes and SNPs was identified using the genome reference consortium human build 37 (GRCh37).

### 2.4. Statistical Analysis

PLINK version 1.90 beta (https://www.cog-genomics.org/plink2, accessed on 3 March 2021) and predictive analytics software (PASW) version 18.0 (SPSS Inc., Chicago, IL, USA) were used for most statistical analyses. We investigated the interaction between SNP and the risk of diseases using logistic regression models with the additive genetic model. The multivariable model was adjusted for age and residence [24,25] to investigate the effect of complex factors. The residential areas initially consisted of 16 areas indicated by administrative district codes. However, we reorganized them into rural (Gyeonggi, Chungcheongbuk, Chungcheongnam, Sejong city, Gangwon, Jeollabuk, Jeollanam, Gyeongsangbuk, and Gyeongsangnam) and urban (Seoul, Busan, Ulsan, Daegu, Daejeon, Incheon, and Gwangju) areas. The association between SNPs and the risk of diseases was computed by the odds ratio (OR) and 95% confidence interval (95% CI). Statistical significance was determined by the two-tailed Student’s *t*-test, and *p*-values < 0.05 were considered significant.

HaploReg database (https://pubs.broadinstitute.org/mammals/haploreg/haploreg.php, accessed on 3 March 2021) was also used to predict the potential functional effects of the *MYLK* rs12163585 and *PTGS1* rs1213265 genotypes. The geography of genetic variants (GGV) browser (https://popgen.uchicago.edu/ggv, accessed on 3 March 2021) was utilized to report the worldwide frequency of the minor alleles of SNPs. 

### 2.5. Ethical Review

This study was approved by the Institutional Review Board of the Korean National Institute of Health (KNIH, KBN-2019-004) and Hoseo University (IRB approval no.: 1041231-150811-BR-034-03). Written informed consent was obtained from all subjects.

## 3. Results

### 3.1. Subject Characteristics

In this study, 18,183 females from the HEXA cohort were included in the association study. Age and the number of subjects with each disease are listed in Table 1. Healthy controls were filtered from 18,183 females in the HEXA cohort and categorized into 4356 healthy controls and 1382 women with osteoporosis (cases). Similarly, there were 3922 healthy controls and 1126 women with benign breast tumors (cases). There were 285 ovariectomy cases in the total HEXA females, and 61 and 45 females had ovariectomies in the healthy controls and cases in the osteoporosis group, respectively. In the benign breast tumor group, 54 healthy controls and 38 subjects with benign breast tumors underwent ovariectomies. The osteoporotic patients were older (average 59.6 years) than the subjects in the control group (average 50.5 years). In addition, the age at ovariectomy in the osteoporotic group was older (average 47 years) than that of the healthy controls (average 45.33 years). There was no significant difference between the cases and the controls in the benign breast tumor group.

### 3.2. Selection of SNPs from Genome-Wide Association Study Based on the HEXA Data

In the GWAS, the SNPs (*p* < 0.001) were filtered based on the HEXA data. Of them, 464 and 469 SNPs associated with benign breast tumor and osteoporosis were found respectively. Only five SNPs were common between the two diseases (rs3732486, rs3732487, rs58154051, rs2293973, and rs1213265). Two (rs3732486 and rs3732487) of the five SNPs were found in the *MYLK* gene, rs58154051 in the *RPS6KA2* gene, rs2293973 in *DLGAP2*, and rs1213265 in *PTGS1* (Table 2). In this study, after excluding genes unrelated to osteoporosis or breast disease, we focused on the *MYLK* and *PTGS1* genes, which presented the least *p*-value and the highest odds ratio for both diseases. The rs3732487 SNP in *MYLK* showed associations with both benign breast tumor (OR = 1.20, 95% CI: 1.09–1.32, *p* = 1.94 × 10^−4^) and osteoporosis (OR = 1.21, 95% CI: 1.09–1.35, *p* = 2.57 × 10^−4^). In addition, rs1213265 in *PTGS1* showed significant associations with benign breast tumors (OR = 1.81, 95% CI: 1.29–2.53, *p* = 6.03 × 10^−4^) and osteoporosis (OR = 2.03, 95% CI: 1.36–3.03, *p* = 4.90 × 10^−4^).

### 3.3. Association of SNPs with Benign Breast Tumor and Osteoporosis

We analyzed the association between benign breast tumors and osteoporosis and *MYLK* and *PTGS1* SNPs. Sixty SNPs in the *MYLK* gene and 12 SNPs in the *PTGS1* gene were found. Among the 60 SNPs in the *MYLK* gene, nine and six SNPs were significantly associated with benign breast tumors and osteoporosis, respectively (Table 3 and Supplementary Table S1). In addition, three common SNPs (rs3732487, rs3732486, and rs12163585) were associated with both diseases. In the case of the *PTGS1* gene, of the 12 SNPs, two common SNPs (rs1213265 and rs3119773) were associated with benign breast tumors and osteoporosis (Supplementary Table S1). The association of these five SNPs in the two genes with benign breast tumors and osteoporosis in the HEXA cohort females was analyzed using the additive model. While the *MYLK* rs3732487 and rs3732486 variants showed similar patterns of increased risk of benign breast tumors (OR = 1.20, 95% CI: 1.09–1.32, *p* = 1.94 × 10^−4^; OR = 1.19, 95% CI: 1.08–1.31, *p* = 3.52 × 10^−4^, respectively) and osteoporosis (OR = 1.21, 95% CI: 1.09–1.35, *p* = 2.57 × 10^−4^; OR = 1.21, 95% CI: 1.09–1.34, *p* = 3.23 × 10^−4^, respectively), the rs12163585 variant was associated with a decreased risk of benign breast tumors and osteoporosis (OR = 0.87, 95% CI: 0.79–0.96, *p* = 5.69 × 10^−3^; OR = 0.88, 95% CI: 0.80–0.98, *p* = 1.98 × 10^−2^, respectively) (Table 3). In the case of the *PTGS1* gene, the rs1213265 and rs3119773 variants significantly increased the risk of benign breast tumors (OR = 1.89, 95% CI: 1.23–2.88, *p* = 3.38 × 10^−3^; OR = 1.88, 95% CI: 1.23–2.88, *p* = 3.41 × 10^−3^, respectively) and osteoporosis (OR = 2.88, 95% CI: 1.36–3.85, *p* = 1.93 × 10^−3^; OR = 2.28, 95% CI: 1.35–3.84, *p* = 1.99 × 10^−3^, respectively) (Table 3). Nine other SNPs in the *MYLK* gene showed association with benign breast tumors or osteoporosis (*p* < 0.05), but the association was significant in only one of the two diseases.

### 3.4. MYLK rs12163585 Variant and Ovariectomy with Osteoporosis

**Figure 2:**
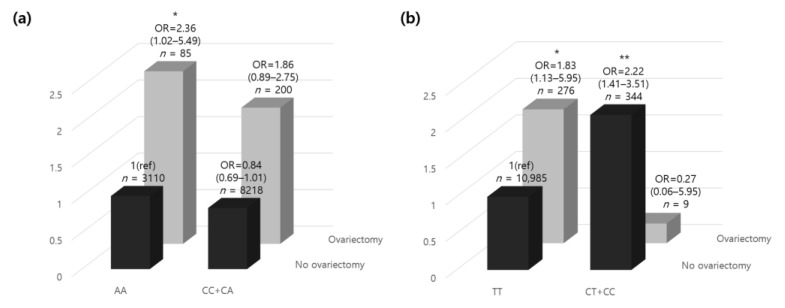
The relative odds ratio of osteoporosis according to oophorectomy and (**a**) *MYLK* rs12163585 and (**b**) *PTGS1* rs1213265 genotypes. The ORs (95% CI) of the genetic correlation between dominant/homozygous genotypes of each gene and ovariectomy are shown. The homozygous model was set as the reference allele. *p*-values were adjusted for age and residential area by analysis of covariance. * *p* < 0.05, ** *p* < 0.01. Abbreviations: OR, odds ratio; CI, confidence interval.

Excluding the participants with missing genotype data (*n* = 16), among the 11,613 participants, there were 200 ovariectomized participants with minor alleles and 85 with homozygous genotypes in the major alleles. In contrast, in the non-ovariectomized participants, the number of minor allele carriers was 8218, and 3110 carried a homozygous genotype in the major allele. In the presence of a minor rs12163585 allele, the risk of osteoporosis was increased 1.86-fold in women who underwent an ovariectomy. However, in individuals carrying the homozygous genotype in the major alleles, the risk of osteoporosis was significantly increased by 2.36-fold (Figure 2a). The results showed that those who underwent ovariectomy, which increased the risk of osteoporosis and was minor allele carriers, carried a decreased risk of developing osteoporosis than those without minor alleles. Those who had minor alleles and underwent ovariectomy had a lower risk of developing osteoporosis than those carrying homozygous genotypes in the major alleles. Our results confirmed that having a minor rs12163585 allele lowered the risk for osteoporosis, which can be interpreted in the same context as the GWA study results.

### 3.5. PTGS1 Variant rs1213165 and Ovariectomy with Osteoporosis

Excluding participants with missing genotype data (*n* = 15), the 11,614 participants included nine minor allele carriers and 276 homozygous genotype major allele carriers who had ovariectomies. In contrast, in the non-ovariectomized participants, the number of minor allele carriers was 344, and 10,985 carried homozygous genotypes in the major allele. With a homozygous rs1213165 genotype in the major allele, the risk of osteoporosis was increased 1.83-fold in women who underwent ovariectomy (Figure 2b). In contrast, in individuals carrying a minor allele, the risk of osteoporosis was decreased 0.27-fold, but there was no statistical significance. In addition, those who did not undergo ovariectomy and were minor allele carriers had a 2.22-fold higher risk of developing osteoporosis than those without minor alleles. Interestingly, those who carried minor alleles and did not undergo ovariectomy had a higher risk of osteoporosis than those with a homozygous genotype in the major allele who underwent ovariectomy.

### 3.6. Functional Annotations of MYLK and PTGS1

The HaploReg database was used to predict the potential functional effects of *MYLK* rs12163585 and *PTGS1* rs1213265 (Supplementary Table S2). Both *MYLK* rs12163585 and *PTGS1* rs1213265 were found to change the motif factor-binding site, as shown in Supplementary Table S2. We also performed the analysis of eQTL for *MYLK* and *PTGS1* based on GTEx databases (Supplementary Figure S1). Gene expression for *MYLK* was high in the female genital organs, especially breast, cervix, ovary, and uterus.

## 4. Discussion

In this prospective GWAS, we identified an association between *MYLK* rs12163585 and *PTGS1* rs1213265 variants, ovariectomy, and the risk of osteoporosis using HEXA Korean women data. Our results showed that (1) women with a homozygous genotype in the *MYLK* rs12163585 major alleles had an increased risk of osteoporosis following ovariectomy than those with minor alleles, and (2) women who had the minor *PTGS1* rs1213265 allele and were not ovariectomized carried a higher risk of osteoporosis than those with homozygous genotype of the major alleles and undergoing ovariectomy. The GWAS of osteoporosis and benign breast tumors revealed that five common SNPs in four genes were significantly associated with the two diseases. SNP rs58154051, located on chromosome 6 and belonging to the *RPS6KA2* gene, did not show a statistically significant relationship with ovariectomy or osteoporosis (Supplementary Figure S2). SNP rs2293973, which is located on chromosome 8 and belongs to *DLGAP2*, was considered a gene variant unrelated to osteoporosis or breast disease. From the present study, following genetic signals, we can prevent osteoporosis and breast cancer, and suggest ovariectomy or not. However, further studies with greater age of cases and large sample size, especially in stratified analysis, are required in the future. To the best of our knowledge, no study has reported the association between ovariectomies and osteoporosis with *MYLK* and *PTGS1* genes until now, and consequently, the *MYLK* and *PTGS1* genes were selected in the present study.

Although the genetic variations in the *MYLK* gene were selected as the targets in our study, its association with breast disease or osteoporosis has yet to be reported. *MYLK* is an element of the actin cytoskeleton and is involved in foundational cellular processes such as cell adhesions, migration, and survival [26]. It is included in the oxytocin signaling pathway and hence, RhoA/Rho kinase pathways are also activated, contributing to the invasion of cancer cells [27]. Expression of *MYLK* is downregulated in breast cancer and loss of *MYLK* leads to disruption of cell–cell adhesion and invasive behavior of breast epithelial cells [28].

Meanwhile, *MLCK* is well known as a molecular target in lung inflammation, a defining feature of sepsis and acute lung injury (ALI) [29]. Gao et al. speculated that *MYLK* was a candidate gene engaged in acute lung injury susceptibility and disease [30]. Another study showed that the thoracic aortic disease phenotype was associated with *MYLK* pathogenic variants [31]. Recently, Dai et al. reported the higher expression of circular RNA (circRNA) MYLK in human prostate cancer tissue and suggested using circRNA-MYLK as a tool to diagnose and determine treatments for prostate cancer [32]. The *MYLK* gene significantly influenced the progression of prostate cancer, which is a sex hormone-dependent disease in males, and the *MYLK* variants found in this study were associated with benign breast tumors related to female hormones, in line with the previous study. Furthermore, androgen, involved in maintaining bone mass density and preventing osteoporosis, is transformed into endogenous estrogen. The ovaries generate enormous amounts of androgen for years in postmenopausal women, helping to retain bone mass. Ovariectomy, which reduces androgen production, may increase the risk of osteoporosis [33]. Our results show the correlation between the *MYLK* gene and ovariectomy and demonstrate that the risk of osteoporosis in women who underwent ovariectomy was significantly higher and nearly double the risk of those without (Figure 2a).

*PTGS1*, also known as cyclooxygenase 1 (*COX1*), is ubiquitously found in tissues and involved in the biosynthesis of prostaglandin (PG), which regulates renal, gastrointestinal, and platelet function [34]. Besides, *PTGS1* has been connected with multiple pathological disorders including inflammation, arthritis, and cancer. One study had compared whole-genome expression data of breast tissue samples with serum hormone levels using data from healthy women and breast cancer patients using microarrays. *PTGS1* was found differentially expressed dependent on estradiol levels, which was downregulated in breast samples from women with high serum estradiol [35]. In both ex vivo and in vivo studies, *PTGS1* that controls osteogenesis of adipose-derived stem cells via regulating the NF-κB signaling pathway is required for the osteogenic differentiation of adipose-derived stem cells [36]. Nagao et al. performed a meta-analysis to determine the association between *PTGS* variants and nonsteroidal anti-inflammatory drugs (NSAIDs), which inhibit the biosynthesis of PG by *PTGS* and reduce inflammation [37]. Another study confirmed wide genomic regions that caused inflammatory arthritis in a heterogeneous [10] mice cohort and identified *PTGS1* as a key candidate based on the differential expression in arthritis [38]. Wang et al. reported that the depletion of *PTGS1* promoted osteogenesis in adipose-derived stem cells and suppressed the NF-κB pathway. Additionally, the knockdown of *PTGS1* may regulate the inflammatory microenvironment during bone remodeling [36].

Most hysterectomies performed for gynecologically benign conditions, preserve the ovaries. However, occasionally, some physicians suggest bilateral ovariectomies (BOs) along with hysterectomy to prevent the development of cancer [39]. The hysterectomy rates for benign disease were 1.48, 1.49, and 1.52/1000 Korean women aged over 16 years in 2007, 2008, and 2009, respectively, which showed an increasing trend [40]. In our present study, we considered a case of ovariectomy when bilateral ovariectomy was performed, and the rate of ovariectomies for benign breast tumors was 3.37/1000 women. The data used in the study were followed up from 2004 to 2013, with increasing trend similar to that of the previous study.

We analyzed the minor allele frequency of *MYLK* rs12163585 and *PTGS1* rs1213265 with a geographic genome variants (GGV) browser. The GGV browser uses maps of allelic frequencies in populations distributed across the globe based on 1000 genomes (hg19). While rs12163585 was mostly seen in Southeast Asia, rs1213265 was mostly detected in Africa (Supplementary Figure S1). The *MYLK* rs12163585 minor allele was associated with a decreased risk of osteoporosis and benign breast tumors in Asia, while the *PTGS1* rs1213265 minor allele detected mostly in Africa was associated with an increase in both diseases. In the Haploview results, *MYLK* rs12163585 was predicted to change the motif factor binding site of GATA (Supplementary Table S2). GATAs (GATA-DNA-binding protein) are known as a transcription factor in osteoblasts and functions in transducing cell survival signaling. GATAs are expressed in osteoblasts and control osteoblast survival and functions [41]. Above all, GATA-3 has been reported that its expression is induced during fracture healing and many studies have reported the correlation between the GATA-3 and estrogen receptor in breast cancers as well [42,43,44]. Additionally, SOX, which was predicted to change the motif factor binding site by *MYLK*, was suggested to sensitive triple-negative breast cancer marker along with GATA-3 [45]. Expression of HAND1, which was predicted to changed motif factor binding site by *PTGS1*, develops long bones and involves in their morphogenesis [46]. *Smad4* gene that has interaction with estrogen receptor α is required for TGF-β-induced epithelial to mesenchymal transition and bone metastasis of breast cancer cells [47,48,49].

## 5. Conclusions

Genetic variants in *MYLK* and *PTGS1* are associated with both benign breast tumors and osteoporosis. Analysis of the differences in the risk of osteoporosis and ovariectomy in the *MYLK* rs12163585 and *PTGS1* rs1213265 genotypes showed significant associations with each genotype. Consequently, pathological factors such as ovariectomies substantially affect the association between gene variants and osteoporosis in Korean women.

## Figures and Tables

**Table 1:** Characteristics of women in the Health Examinee (HEXA) study cohort.

Quantitative Trait Analysis	Characteristics	Case-Control Analysis for Benign Breast Tumor		
		Controls	Cases	*p*-Value *
18,183	No.	3922	1126	
53.14 ± 7.62	Age (M years ± SD)	49.92 ± 6.96	52 ± 6.69	<0.001
		**Case-control analysis for osteoporosis**		
		**Controls**	**Cases**	*p*-value *
	No.	4356	1382	
	Age (M years ± SD)	50.75 ± 7.48	59.59 ± 6.38	<0.001

Abbreviations: M, mean value; SD, standard deviation. * Significant differences in characteristics between the controls and the cases were determined via two-tailed Student’s *t*-test.

**Table 2:** Significant association of SNPs in both benign breast tumors and osteoporosis in the HEXA women cohort.

Gene	Chr	SNP	Minor Allele	MAF	Function	Benign Breast Tumor (Case = 1126, Control = 3922)		Osteoporosis (Case = 1382, Control = 4356)	
						OR (95% CI)	*p* Value	OR (95% CI)	*p* Value
*MYLK*	3	rs3732486	A	0.487	intron	1.19 (1.08~1.31)	3.52 × 10^−4^	1.21 (1.09~1.34)	3.23 × 10^−4^
	3	rs3732487	G	0.489	intron	1.20 (1.09~1.32)	1.94 × 10^−4^	1.21 (1.09~1.35)	2.57 × 10^−4^
*RPS6KA2*	6	rs58154051	A	0.208	intron	1.23 (1.09~1.37)	4.20 × 10^−4^	1.25 (1.10~1.41)	6.51 × 10^−4^
*DLGAP2*	8	rs2293973	A	0.185	intron	0.80 (0.71~0.91)	6.62 × 10^−4^	0.78 (0.68~0.89)	2.78 × 10^−4^
*PTGS1*	9	rs1213265	C	0.015	intron	1.81 (1.29~2.53)	6.03 × 10^−4^	2.03 (1.36~3.03)	4.90 × 10^−4^

Age and residential area were included as covariants in all genetic models. Abbreviations: Chr, Chromosome; MAF, minor allele frequency; OR, odds ratio; CI, confidence interval. This analysis yielded 5 SNPs demonstrating genome-wide significance (*p*-value < 0.001), including *MYLK* and *PTGS1* genes, which were examined from this study.

**Table 3:** Association of SNPs in the *MYLK* and *PTGS1* genes with benign breast tumor and osteoporosis in the HEXA women included in the additive genetic model.

Gene	SNP	Minor Allele.	MAF	Function	Benign Breast Tumor		Osteoporosis	
					OR (95% CI)	*p* Value	OR (95% CI)	*p* Value
*MYLK*	rs3732487	G	0.489	missense	1.2 (1.09~1.32)	1.94 × 10^−4^	1.21 (1.09~1.35)	2.57 × 10^−4^
	rs3732486	A	0.487	missense	1.19 (1.08~1.31)	3.52 × 10^−4^	1.21 (1.09~1.34)	3.23 × 10^−4^
	rs12163585	C	0.475	intron	0.87 (0.79~0.96)	5.69 × 10^−3^	0.88 (0.80~0.98)	1.98 × 10^−2^
	rs58036435	T	0.468	intron	0.88 (0.80~0.97)	1.17 × 10^−2^	0.91 (0.82~1.01)	0.066
	rs3796164	G	0.438	intron	0.89 (0.80~0.97)	1.26 × 10^−2^	0.93 (0.84~1.30)	0.190
	rs151091281	T	0.043	intron	0.74 (0.57~0.96)	2.29 × 10^−2^	0.87 (0.67~1.13)	0.300
	rs77820417	A	0.428	intron	0.91 (0.82~1.00)	4.33 × 10^−2^	0.91 (0.82~1.00)	0.060
	rs9836287	C	0.035	intron	1.28 (1.00~1.63)	4.71 × 10^−2^	1.05 (0.80~1.39)	0.711
	rs117430366	C	0.029	intron	0.74 (0.54~1.00)	4.84 × 10^−2^	0.86 (0.63~1.16)	0.319
	rs181117066	G	0.010	intron	1.27 (0.78~2.07)	0.339	1.99 (1.21~3.29)	6.89 × 10^−3^
	rs74629231	C	0.010	intron	1.29 (0.23~0.82)	0.268	1.88 (1.17~3.01)	8.77 × 10^−3^
	rs7637909	C	0.047	intron	0.89 (0.12~0.71)	0.267	0.76 (0.59~0.98)	3.22 × 10^−2^
*PTGS1*	rs1213265	C	0.015	intron	1.81 (1.29~2.53)	6.03 × 10^−4^	2.03 (1.36~3.03)	4.90 × 10^−4^
	rs3119773	A	0.015	intron	1.88 (1.23~2.88)	3.41 × 10^−3^	2.28 (1.35~3.84)	1.99 × 10^−3^
	rs12555242	C	0.025	intron	1.81 (1.29~2.53)	6.01 × 10^−4^	1.11 (0.80~1.55)	0.538

## Data Availability

The data presented in this study are available on request from the corresponding author. The data are not publicly available due to ethnical concerns.

## Supplementary Materials

The following are available online at https://www.mdpi.com/2073-4425/12/3/378/s1, Figure S1: Geographic distribution of (a) *MYLK* rs12163585 and (b) *PTGS1* rs1213265. The position of the SNPs is shown at the top (chr3:123416351 and chr9:125136447) and the blue quarter indicates a 0.25 minor allele global frequency based on 1000 genomes (hg19). In Korea, the minor allele frequencies of rs12163585 and rs1213265 were 0.48 and 0.015, respectively. Figure S2: Relative odds ratio of osteoporosis according to the ovariectomy and *RPS6KA2* rs58154051. The OR (95% CI) of genetic interaction of dominant /homozygous genotypes of each gene with variable ovariectomy. The homozygous model was set as the reference allele. p values were adjusted for age and residential area by analysis of covariance. Abbreviations: OR, odds ratio; CI, confidence interval. Table S1: Association of the SNPs in *MYLK* and *PTGS1* with benign breast tumors and osteoporosis in the HEXA women cohort in the additive genetic model. Table S2: Results of HaploReg analysis of *MYLK* rs12163585 and *PTGS1* rs1213265. Abbreviations: A1, minor allele; A2, major allele; IPSC, Ips DF 6.9 cells.
