# Supplementary figures and images for: MYLK and PTGS1 Genetic Variations Associated with Osteoporosis and Benign Breast Tumors in Korean Women
Source: Genes (Basel). 2021 Mar 6;12(3):378. doi: 10.3390/genes12030378 (PMC7998336; doi:10.3390/genes12030378)

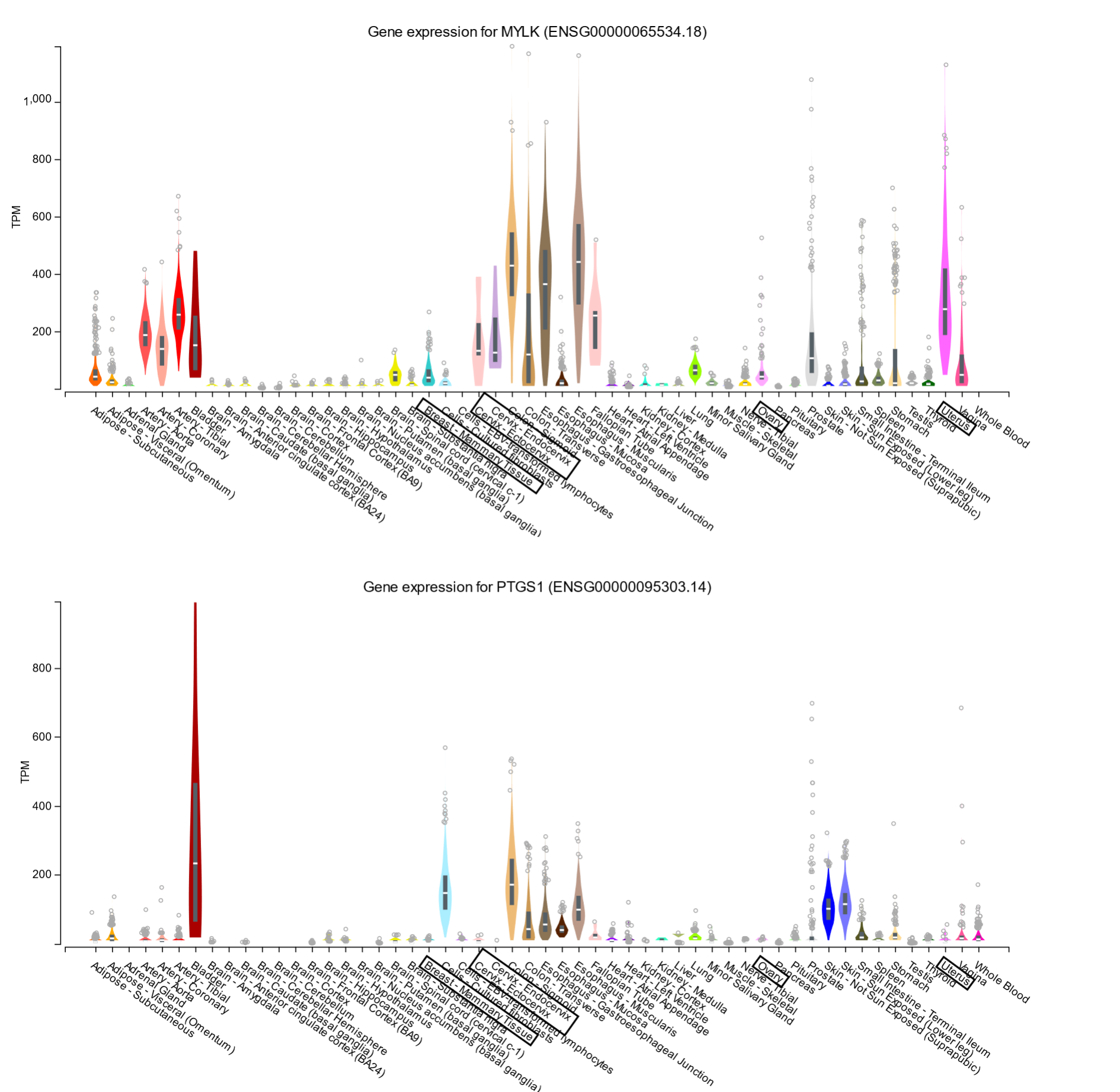

Supplement: Supplementary file 1 [file genes-12-00378-s001.zip › genes-1091262 supplementary/Supplementary Figure S1_600 dpi.tif]

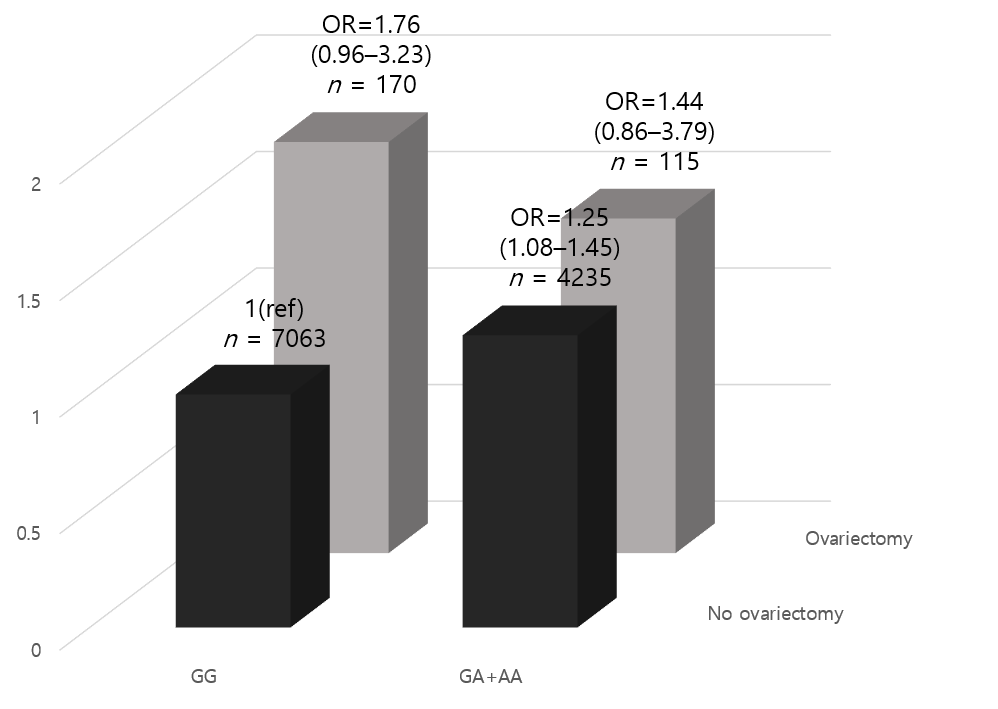

Supplement: Supplementary file 1 [file genes-12-00378-s001.zip › genes-1091262 supplementary/Supplementary Figure S2.tif]
